# Polymorphism of the growth hormone gene *GH1* in Polish children and adolescents with short stature

**DOI:** 10.1007/s12020-020-02305-5

**Published:** 2020-04-27

**Authors:** Katarzyna Anna Majewska, Andrzej Kedzia, Przemyslaw Kontowicz, Magdalena Prauzinska, Jaroslaw Szydlowski, Marek Switonski, Joanna Nowacka-Woszuk

**Affiliations:** 1 Department of Clinical Auxology and Pediatric Nursing, Poznan University of Medical Sciences, Szpitalna 27/33, Poznan, Poland; 2 Department of Genetics and Animal Breeding, Poznan University of Life Sciences, Wolynska 33, 60-637 Poznan, Poland; 3 Department of Pediatric Otolaryngology, Poznan University of Medical Sciences, Szpitalna 27/33, 60-572 Poznan, Poland

**Keywords:** *GH1* gene, Mutation, Sequencing, SNP, Growth hormone deficiency (GHD), Idiopathic short stature (ISS)

## Abstract

**Purpose:**

Short stature in children is a significant medical problem which, without proper diagnosis and treatment, can lead to long-term consequences for physical and psychological health in adult life. Since human height is a polygenic and highly heritable trait, numerous variants in the genes involved in growth—including the growth hormone (*GH1*) gene—have been identified as causes of short stature.

**Methods:**

In this study, we performed for the first time molecular analysis of the *GH1* gene in a cohort (*n* = 186) of Polish children and adolescents with short stature, suffering from growth hormone deficiency (GHD) or idiopathic short stature (ISS), and a control cohort (*n* = 178).

**Results:**

Thirteen SNP variants were identified, including four missense variants, six in 5′UTR, and three in introns. The frequency of minor missense variants was low (<0.02) and similar in the compared cohorts. However, two of these variants, Ala39Val (rs151263636) and Arg42Leu (rs371953554), were found (heterozygote status) in only two GHD patients. These substitutions, according to databases, can potentially be deleterious.

**Conclusions:**

Mutations of *GH1* causing short stature are very rare in the Polish population, but two potentially causative variants need further studies in a larger cohort of GHD patients.

## Introduction

Human growth is a complex process regulated by multiple factors—genetic, hormonal, and environmental. However, its coefficient of heritability (*h*^2^) is very high at over 0.8 [1]. Thus, polymorphisms of the genes associated with growth are considered important causes of short stature. Growth hormone is a major hormone involved in linear growth, although it also influences the body’s general metabolism. It is secreted by the anterior lobe of the pituitary gland, mainly during sleep at night, and its efficient release is crucial for the normal course of growth in children. The main symptoms of isolated growth hormone deficiency (GHD) are short stature and poor growth velocity [2–4].

The definition of short stature is based on statistical data, assuming that the normal values for the population are within the range of ±2 standard deviations (SDs), adjusted for age and gender. A child whose height is below −2SD is thus considered to be of short stature [5]. Many of these children show no signs of any other disease and simply demonstrate a constitutionally delayed process of growth and maturation. In some children, however, disorders in the growth process are caused by specific clinical conditions, such as chronic infectious diseases, kidney or liver failure, congenital heart defects, intestinal malabsorption, thyroid dysfunction, or genetic syndromes such as Down’s syndrome and Turner’s syndrome [3–5].

Short stature disturbs the functional and psychosocial abilities of a child, and even more so of an adult. In the case of abnormal growth, safe and effective therapeutic intervention is possible only in the developmental period, before growth ends, which occurs around the bone age of 14 in girls, and 16 years in boys, but in many short children bone maturation is substantially delayed. Correct early diagnosis and appropriate treatment are essential for effective growth improvement and to prevent the consolidation of problems in adulthood [6, 7]. Thus, recognition of the etiopathogenesis is important for diagnostic and therapeutic process.

The congenital form of GHD as a cause of short stature occurs with an incidence ranging from 1 in 4000 to 1 in 10,000 live births. It may be caused by genetic factors or structural changes in the hypothalamus and pituitary [2, 5, 8, 9], but the most commonly diagnosed form is idiopathic. Familial GHD may affect from 3 to 30% of cases, depending on the population studied [2, 9]. Idiopathic short stature (ISS) is diagnosed when growth hormone secretion, as evaluated in laboratory tests, is normal and no other cause for growth disorders has been found [10]. However, positive results of laboratory tests do not exclude abnormalities in growth hormone structure that lead to disorders in its hormonal activity.

There are numerous genes that control growth, variants of which can contribute or cause short stature [11]. Growth hormone (GH) plays a crucial role among these as a major regulator of growth, and its variants may cause GHD, leading to short stature [12–15]. However, it should be pointed out that short stature can be caused by variants of other genes (such as *GHRHR*, *POU1F1*, *PROP1*, and *IGF1*) and other mechanisms, including chromosomal abnormalities, copy number variation polymorphisms, and disrupted genomic imprinting [12]. The aim of this study was to seek an association between variants of *GH1* gene and short stature in Polish children and adolescents.

## Material and methods

The study population consisted of a cohort of 186 children (136 boys, including 111 with GHD and 25 with ISS; and 50 girls, including 38 with GHD and 12 with ISS) with short stature from central and western Poland, aged 3–16 years, who were patients of Karol Jonscher Teaching Hospital, Poznan University of Medical Sciences (Table 1). The mean age of patients was 9.5 (boys) and 9.3 years (girls). For two patients, blood samples from both parents and brothers were also collected.

**Table 1:** Characteristics of the studied cohorts

Parameter	Patients *n* = 186						Controls *n* = 178	
	Boys			Girls			Boys *n* = 96	Girls *n* = 82
	GHD *n* = 111	ISS *n* = 25	Together *n* = 136	GHD *n* = 38	ISS *n* = 12	Together *n* = 50	–	–
Age in years (mean ± S.D.)	9.57 ± 3.41	9.45 ± 3.19	9.55 ± 3.37^a^	9.21 ± 2.69	9.56 ± 2.35	9.30 ± 2.61	8.57 ± 3.82^a^	9.49 ± 4.15
hSDS (mean ± S.D.)	−2.84 ± 0.66	−2.76 ± 0.78	−2.83 ± 0.68^B^	−2.56 ± 0.52	−2.67 ± 0.34	−2.58 ± 0.49^C^	0.31 ± 0.89^B^	0.22 ± 0.88^C^
mpSDS (mean ± S.D.)	0.35 ± 0.81^#^	0.26 ± 0.75	0.34 ± 0.80^§, D^	0.35 ± 0.66^#^	0.36 ± 0.52	0.35 ± 0.63^§, E^	1.09 ± 0.71^D^	0.95 ± 0.67^E^

Means with the same letter differ statistically: small letter *P* < 0.05; capital letter *P* < 0.001

*GHD* growth hormone deficiency, *ISS* idiopathic short stature, *S.D.* standard deviation, *hSDS* height standard deviation score, *mpSDS* midparental height standard deviation score

^#^mpSDS was calculated for 110 boys and 37 girls, as no information was available on biological parents for two patients

^§^mpSDS was calculated for 135 boys and 49 girls in total

All patients underwent a physical examination (measured height and assessed general health), and had estimated growth hormone secretion based on physiological sleep test and two pharmacological stimulation tests (with insulin, clonidine, or glucagone). Height measurements were performed using a stadiometer (Holtain), in centimeters (cm) with an accuracy of 1 mm. Children with genetic syndromes and chronic diseases, such as multiple pituitary hormone deficiency, or malabsorption, were not included in the study. Patients with levels of GH sleep-associated secretion and in stimulation tests below 10 ng/ml (DIAsource hGH-IRMA), were diagnosed with GHD. This criterium was applied for both, boys and girls. No cause of growth disorders was found in the ISS children. The control cohort included 178 children, of which 96 were boys and 82 girls (mean age was 8.6 years for boys and 9.5 years for girls), with normal height and presenting normal development, aged 3.3–17 years and admitted for other diagnostic or therapeutic purposes to the hospital. Additional information collected on patients and controls included body height of both parents. The midparental height standard deviation score (mpSDS) and children’s height standard deviation score (hSDS) were calculated using the collected data. The study protocol was approved by the local Ethics Committee at Poznan University of Medical Sciences (No. 1070/15). The legal guardians of all participants gave their informed consent. Statistical analysis included Student’s *t* test for means in two unrelated groups (https://www.socscistatistics.com/tests/studentttest/default2.aspx).

DNA for molecular analysis was isolated from blood samples using a DNA Blood Mini Kit (A&A Biotechnology), and DNA quantity was determined on a Nanodrop spectrophotometer (Thermo Fisher Scientific). PCR amplification was conducted under standard conditions; details of primer sequences are given in Table S1. Amplicons were enzymatically purified by alkaline phosphatase and exonuclease I (Thermo Fisher Scientific) following amplification with BigDye Terminator v3.1 Cycle Sequencing Kit (Thermo Fisher Scientific) and filtration on a Sephadex G50 (Sigma). Next, capillary electrophoresis was run on a Genetic Analyzer 3130 (Applied Biosystems). Data were analyzed using DNAStar software. Minor allele frequency (MAF) was calculated for all identified polymorphisms, while differences between studied cohorts were analyzed using the odds ratio test (https://www.medcalc.org/calc/odds_ratio.php). The MAF below 1% for the sequence variant can be considered as rare variants while the MAF above this 1% is thought as common polymorphism. Provean Protein software was used to predict the effect of amino acid substitution in the GH1 protein sequence (http://provean.jcvi.org/seq_submit.php).

## Results

The calculated means for hSDS in the patient group were −2.83 for boys and −2.58 for girls, while the values for controls were 0.31 and 0.22 for boys and girls, respectively. These differences were statistically highly significant (*P* < 0.001). The mpSDS in the patient cohort differed significantly from the controls and was 0.34 and 0.35 for boys and girls, respectively; in the controls it was 1.09 for boys and 0.95 for girls.

Sequence analysis was performed for the entire coding sequence of *GH1* (5 exons, 770 bp), 5′-untranslated region—5′UTR (194 bp), 3′-flanking sequence—3′UTR (109 bp), and short intronic fragments in the vicinity of the exons. Altogether, 13 single nucleotide polymorphisms (SNPs) were found (Table 2). Six of them were located in 5′ UTR, four in the coding sequence (missense substitutions), and three in introns. The MAF was calculated and was low (<0.1) for the majority of polymorphic variants, with an exception of rs6171 located in 5′UTR (Table 2). This substitution (T > C) was widely and evenly distributed in patients and control cohorts (MAF of 0.470 and 0.438, respectively). Moreover, multiallelic variation was also observed for the rs695 located in 5′UTR. The majority of patients and controls had a T to A substitution, but four patients with GHD and a single control showed a T > G exchange (Fig. S1). The odds ratio test showed no association between the identified polymorphisms and the occurrence of short stature (Table 2).

**Table 2:** Polymorphisms of the *GH1* gene identified in cohorts

SNP ID	Substitution and location in the gene	Amin acid substitution	GHD (*n* = 149)			ISS (*n* = 37)			Controls (*n* = 178)			MAF		*P* value (odds ratio value)
			No of genotypes			No of genotypes			No of genotypes			Patients GHD + ISS (*n* = 186)	Controls (*n* = 178)	
			1/1	1/2	2/2	1/1	1/2	2/2	1/1	1/2	2/2			
rs6171	T > C in 5′UTR	–	36	85	28	15	10	12	56	88	34	0.470	0.438	0.383 (0.878)
rs695	T > A^a^ in 5′UTR	–	121	22	2	29	8	0	145	31	1	0.093^b^	0.093^b^	0.993 (0.998)
rs6175	C > G in 5′UTR	–	145	4	0	37	0	0	176	2	0	0.011	0.006	0.452 (0.520)
rs9282699	T > C in 5′UTR	–	139	9	1	35	2	0	166	12	0	0.035	0.034	0.927 (0.963)
rs6172	T > G in 5′UTR	–	139	9	1	35	2	0	167	11	0	0.035	0.031	0.760 (0.881)
rs6173	A > C in 5′UTR	–	138	11	0	36	1	0	164	14	0	0.032	0.039	0.608 (1.228)
rs2001345	T > C in exon 1	p.Thr3Ala	144	5	0	35	2	0	176	2	0	0.019	0.006	0.129 (0.295)
rs151263636	G > A in exon 2	p.Ala39Val	148	1	0	37	0	0	178	0	0	0.003	0.000	0.518 (0.347)
rs371953554	C > A in exon 2	p.Arg42Leu	148	1	0	37	0	0	178	0	0	0.003	0.000	0.518 (0.347)
rs41295031	C > G in intron 2	–	144	5	0	37	0	0	178	0	0	0.013	0.000	0.109 (0.094)
rs41295053	C > T in intron 2	–	148	0	1	36	1	0	174	4	0	0.008	0.011	0.663 (1.398)
rs5388	C > T in exon 4	p.Val136Ile	146	3	0	36	1	0	174	4	0	0.011	0.011	0.956 (1.040)
rs41295245	G > A in intron 4	–	132	17	0	32	4	1	163	15	0	0.062	0.042	0.225 (0.668)

1: reference variant; 2: alternative variant

*SNP* single nucleotide polymorphism, *UTR* untranslated region, *GHD* growth hormone deficiency, *ISS* idiopathic short stature, *MAF* minor allele frequency

^a^The substitution T > G was found in a heterozygote status for four patients with GHD and one control

^b^MAF was calculated for 182 patients and 177 control

**Fig. 1:**
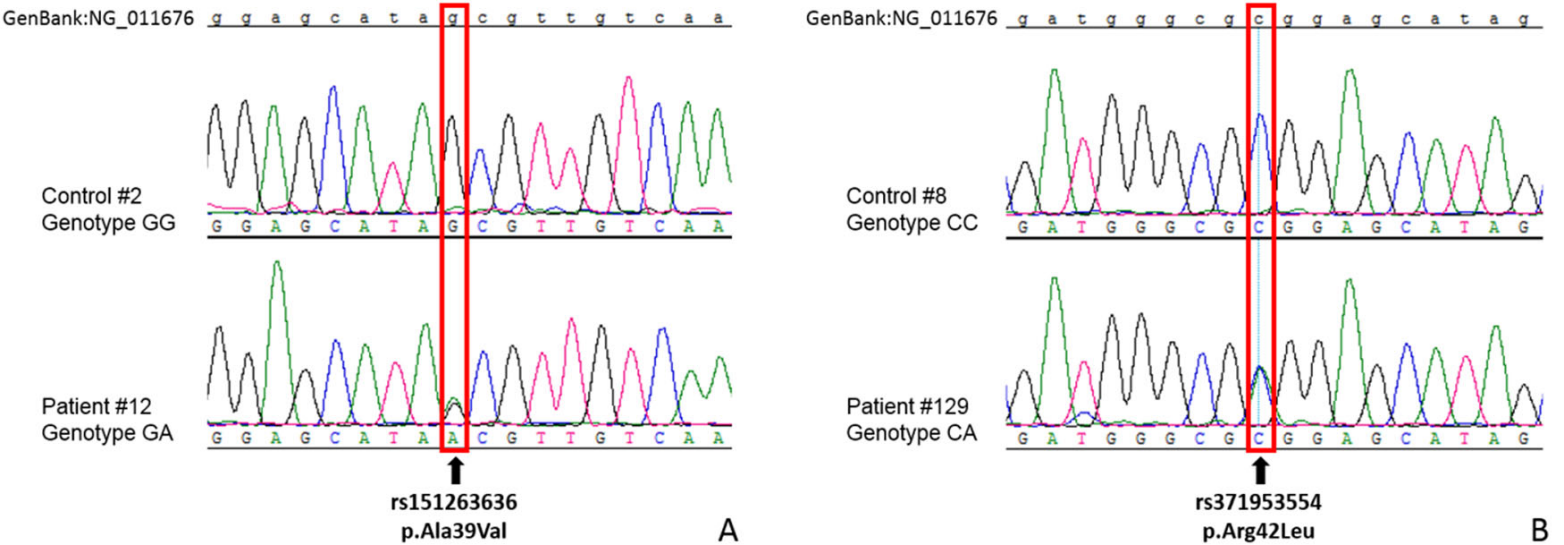
Sequencing of missense variants (in red frames) in exon 2 of *GH1* gene: **a** rs151263636, and **b** rs371953554 in two different GHD patients

Four missense SNPs identified in the coding region have already been described (rs2001345, rs151263636, rs371953554, and rs5388). The first of these, T > C in exon 1, results in a threonine to alanine substitution at the third position of amino acid chain (p.Thr3Ala) and was found in seven heterozygous patients (five with GHD and two with ISS) and in two controls. The next variant, located in exon 2 (rs151263636) and resulting in the substitution of alanine by valine (p.Ala39Val), was observed only in a single heterozygous patient with GHD (Fig. 1a). Similarly, the variant rs371953554, also located in exon 2 and causing an Arg42Leu substitution, was found in a single GHD patient in a heterozygote status (Fig. 1b). The final missense substitution (rs5388), occurring in exon 4, was responsible for a Val136Ile change. The isoleucine variant was identified in three patients with GHD and one with ISS, as well as in four controls (always heterozygotes). Interestingly, Provean Protein software described the Thr3Ala and Val136Ile substitutions are neutral, but the Ala39Val and Arg42Leu alterations as deleterious. The remaining substitutions, located in 5′UTR and introns, were not localized in sites that could disrupt transcription, translation, or splicing events.

**Fig. 2:**
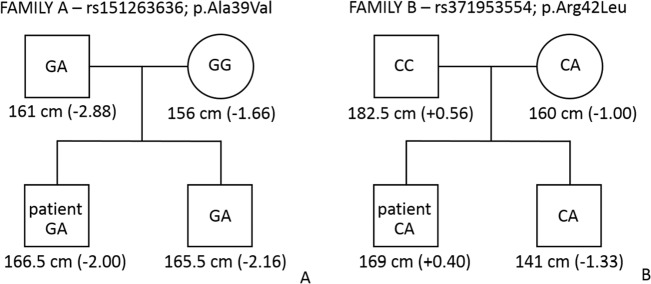
Pedigrees of the studied families in which rs151263636 (**a**) and rs371953554 (**b**) variants segregated. The genotypes are given in a square or circle—according to sex. Heights in cm and height standard deviation score (in brackets) are given for all members. Patient heights were measured after growth hormone treatment

For two SNPs (rs151263636 and rs371953554) found exclusively in two patients, a family study was performed. In family A, where the p.Ala39Val substitution was found, sequencing results showed that this variant was also present in a heterozygote status in the father and a brother (Fig. S2A). The patient (#12) was diagnosed with GHD and started growth hormone therapy at the age of 14, when his height was 140 cm (below the third percentile; hSDS = −3.10). After 3 years of treatment, at the age of 17, his height was 166.5 (at the third percentile; hSDS = −2.00). The patient’s mother is 156 cm in height (between the third and tenth percentiles; hSDS = −1.66), his father is 161 cm tall (below the third percentile; hSDS = −2.88), while his brother is 165.5 cm (below the third percentile; hSDS = −2.16) (Fig. 2a). In family B, the Arg42Leu substitution was found in patient #129 (GHD), as well as in his mother and brother (who were heterozygotes) (Fig. S2B). This patient started growth hormone therapy at the age of six, when his height was 107 cm (below the third percentile; hSDS = −2.88). After eight years of treatment, at the age of 14, his height was 169 cm (between the 50th and 75th percentiles; hSDS = +0.40). His mother is 160 cm tall (between the 10th and 25th percentiles; hSDS = −1.00), his father is 182.5 cm (at the 75th percentile; hSDS = +0.56), while his brother, aged 11.5 years, is 141 cm tall (at the tenth percentile; hSDS = −1.33) (Fig. 2b). However, it must be stated that the brother at the age of 3 was 91 cm tall (at the third percentile; hSDS = −2.0), and in following years, excessive weight gain caused overweight (current body mass index is 23.6, between the 90th and 97th percentiles), but also may have accelerated his growth. All family members in the two pedigrees were of the Caucasian race, had proportional body structure, and no dysmorphic features.

## Discussion

Gaining an understanding of the causes of short stature is essential to describe the complete characteristics of affected patients. It would also make it possible to estimate the risk of another child with this problem being born in a family, and to plan early, appropriate diagnostics and treatment.

To the best of our knowledge, this study presents the first analysis of *GH1* sequence variation in Polish children and adolescents with isolated GHD and ISS. Our analysis revealed thirteen known SNPs, including four variants causing amino acid substitutions in the encoded polypeptide. There have been earlier studies on the association of some of these substitutions with short stature. The rs2001345 (p.Thr3Ala in exon 1) polymorphism was described by Miyata et al. [16] in a cohort of Japanese isolated GHD patients, but it was not indicated that this variant could be causative. In our study, the minor variant (Ala) was found in seven patients with short stature (five with GHD and two with ISS) and in two controls, and its frequency did not differ significantly in the compared cohorts. We can thus confirm the earlier suggestion that this polymorphism is not associated with short stature.

The effect of the next substitution (rs151263636 and p.Ala39Val), included in different databases (e.g., dbSNP and ClinVar), is described as benign or uncertain. We identified the minor variant (Val) in only a single patient. This variant is also very rare in European population—0.004%, (GnomAD database, https://gnomad.broadinstitute.org/). According to Provean Protein software, p.Ala39Val substitution is predicted as deleterious, in spite of the fact that both amino acids (alanine and valine) belong to the same nonpolar, hydrophobic amino acid group. Interestingly, another substitution at the same amino acid position (39) (p.Ala39Thr) was reported by Sundralingam et al. [14], who suggested that its significance was uncertain. Our study of family A showed that the minor variant was inherited by the patient and his brother (who also suffered from short stature, but was not diagnosed earlier) from the father, who was also short (−2.88 hSDS). Thus, the potential significance of this polymorphism cannot be ruled out.

The third variant (rs371953554, p.Arg42Leu) found in the second exon was also observed in a single GHD patient. Another substitution in the amino acid chain at this position (p.Arg42Cys) is described in the available public databases (e.g., dbSNP and UniProtKB). The identified here substitution can be considered potentially deleterious, since arginine is a positively charged amino acid, while leucine is nonpolar and hydrophobic. This could therefore contribute to the short stature phenotype of the patient. The minor variant was also found in his mother (hSDS = −1.00) and in an overweight brother (hSDS ranging between −2.00 at age 3 and −1.33 at 11.5 years). Its frequency is also very low in different populations, including European (0.002%, GnomAD database).

The final missense polymorphism (rs5388), which we found in four patients and four controls, was located in exon 4 (p.Val136Ile), and our in silico study predicted the effect as benign, because valine and isoleucine are both nonpolar hydrophobic amino acids. Interestingly, the valine to phenylalanine substitution at the same position (p.Val136Phe) has previously been described [14, 17]; it was classified as likely to be pathogenic, since phenylalanine is a nonpolar aromatic amino acid.

Familial isolated GHD cases are of four types: (I) autosomal recessive type 1A, caused mainly by deletions of the fragments from 6.5 to 45 kb overlapping the *GH1* gene, or by frameshift deleterious mutations; (II) autosomal recessive type 1B caused by splice site, frameshift nonsense mutations in *GH1*, or variants in growth hormone releasing hormone receptor (*GHRHR*); (III) autosomal dominant type II, mostly caused by *GH1* missense mutations or by variants, leading to splicing disruption in this gene; and (IV) X-linked type III, caused by mutations in *BTK* and *SOX3* genes [18]. Many of the deleterious mutations of *GH1* concern deletions, frameshift variants, and splicing mutations leading to exon skipping, and in consequence to secretion of a shortened GH protein [19]. We found four missense variants in our study; it is known that such alterations can contribute to two types of GHD: autosomal recessive type 1B and autosomal dominant type II [18]. Two of these variants (rs2001345 and rs5388) seem to be neutral. Classification of the other two variants (rs151263636 and rs371953554) needs further study, since they can cause GHD type 1B.

Recent studies of the distribution of *GH1* polymorphisms in GHD patients have revealed several variants that could affect the functionality of the encoded hormone. Babu et al. [20] studied 103 GHD Italian patients and identified four missense SNPs in exon 3: c.261C > T (p.Pro87Pro), c.272A > T (p.Glu91Val), c.255G > A (p.Pro85Pro), and c.246G > C (p.Glu82Asp). In silico analysis predicts that the c.255 and c.272 variants were most likely located in two putative novel exon splicing enhancers, which could cause secretion of the shortened polypeptide (17.5-kDa isoform). This assumption has been confirmed by in vitro studies where rat pituitary cells were transfected with expression vector covering either the wild type or a mutated variant of *GH1* gene. RT-PCR showed that the mutated variant was responsible for greater production of the aberrant GH protein [20]. In the study of Cabrera-Salcedo et al. [21], a known *GH1* variant (p.Arg183His) leading to decreased GH hormone secretion was analyzed in a North-American family with GHD. In this four-generation family, the height SDS of the GHD members varied from −3.8 to −1.9. The same variant was also examined by Pérez-Millán et al. [22], who sequenced 30 known and 37 candidate genes for congenital hypopituitarism and GHD in the exosomes of Argentinian patients. They found the Arg183His polymorphism, described by the authors as p.Arg209His (c.626G > A, ENST00000323322), in a three-generation pedigree with GHD.

Extensive studies conducted in UK and Dutch populations on isolated GHD showed the importance of causative mutations or associated polymorphisms in *GH1* and *GHRHR* genes, especially. In a study of 226 GHD patients in the UK, ten variants (deletion, nonsense, missense, and splice site variants) in the *GH1* gene were identified in 7.4% of patients. Moreover, five missense variants in *GHRHR* gene were also found in 3.7% of patients [23]. In a Dutch cohort of 89 patients, five *GH1* variants (missense and splice sites variants) were diagnosed in 9% of the patients [24]. A broad study of the *GHRHR* gene was undertaken by Cohen et al. [25], who studied 312 French patients with GHD, but who lacked the *GH1* variant. They identified a total of 22 variants (17 novel) in *GHRHR* in 26 patients, including splice site and frameshift variants, intronic deletions, and missense and nonsense substitutions. Detailed analysis of the identified *GHRHR* variants showed that twenty variants were defined as highly probable disease-causing (loss-of-function) mutations, while the effect on protein function was uncertain for another two variants. This study has shown that variants in the *GHRHR* gene are a second cause of GHD, besides *GH1* variants [25].

It should be pointed out that the GHD can also be caused by variants in the genes encoding early or late transcription factors (*PROP1* and *POU1F1*), as well as other genes [9, 26, 27]. However, it should be emphasized that variants of these genes, unlike in the case of GHD caused by *GH1* or *GHRHR* variants, are usually associated with deficiencies of other pituitary hormones, such as prolactin, thyroid stimulating hormone, or even gonadotropins (LH, FSH) [19]. Patients with combined pituitary dysfunction were not included in our study. A broad study of the genetic background of growth deficiencies was also performed in the ISS group. Ten candidate genes were screened using next generation sequencing (NGS) and 18 rare variants (all heterozygote) in the *ACAN*, *FGFR3*, *GHRHR*, *GHR*, *STAT5B*, *IGFALS*, and *IGF1R* genes were found in nineteen patients [10]. A very powerful molecular tool for the identification of candidate genes is genome wide association study (GWAS). Such approach was applied in case-control study of ISS patients and several variants were indicated as associated with the short stature [28]. Since several genes can contribute to this phenotype also oligogenic model of inheritance should be also taken into consideration, as it was shown for isolated gonadotropin-releasing hormone deficiency [29]. The authors proposed the oligogenic model for 2.5% of the patients.

Additional clinical observation of our study was the male predominace among both, children with ISS, and those diagnosed with GHD. It is consistent with numerous studies worldwide [30, 31]. As short stature is diagnosed in both, girls and boys in the same way (hSDS below −2), similar numbers of girls and boys fulfill the criteria of short stature. Male predominance may be a result of general social acceptance for short girls and women, but not boys and men. Girls are historically underinvestigated, while according to current guidelines, they should be evaluated and receive appropriate treatment similarly as boys [32].

## Conclusion

Our study has shown that *GH1* is quite polymorphic in Polish children and adolescents, but that the majority of minor variants are rare, and no significant association with the short stature could be observed. However, two missense substitutions (rs151263636 and rs371953554), identified in two patients and their families, indicate that their contribution to the short stature phenotype is possible. We suggest that whole genome approaches (e.g., GWAS, NGS, or array Comparative Genome Hybridization) should be applied in further searching for the causative or associated variants (SNPs, indels and CNVs) in Polish population. Moreover, multigenerational family studies of the candidate variants are recommended.

## Supplementary material

Supplementary Material
