# Supplementary material for: Polymorphism of the growth hormone gene GH1 in Polish children and adolescents with short stature
Source: Endocrine. 2020 Apr 27;69(1):157–64. doi: 10.1007/s12020-020-02305-5 (PMC7343724; doi:10.1007/s12020-020-02305-5)
Supplement: Supplementary file 1 — Supplementary Material [file 12020_2020_2305_MOESM1_ESM.docx]

**Supplementary material**

**Polymorphism of the growth hormone gene *GH1*** **in Polish children and adolescents with short stature**

Katarzyna Anna Majewska, Andrzej Kedzia, Przemyslaw Kontowicz, Magdalena Prauzinska, Jaroslaw Szydlowski, Marek Switonski, Joanna Nowacka-Woszuk

**Table S1** PCR primer sequences, their location in the *GH1* gene and amplicon length (according to GenBank: NG_011676)

| **Fragment** | **Primer sequences** | **Amplicon length** |
| --- | --- | --- |
| exon 1 and 5’UTR | F: 5’ cacaaccctcacaacactgg  R: 5’ ggccaaatactgggcttaca | 418 bp |
| exon 2 | F: 5’ cgccatgtaagcccagtatt  R: 5’ ggaaaaaccctgagctcctt | 440 bp |
| exon 3 and 4 | F: 5’ cagatgagcacacgctgagt  R: 5’ agagggcagcagtgtttctc | 547 bp |
| exon 5 | F: 5’ gaatcctccaggcctttctc  R: 5’ ccagcttggttcccaataga | 539 bp |

UTR – untranslated region


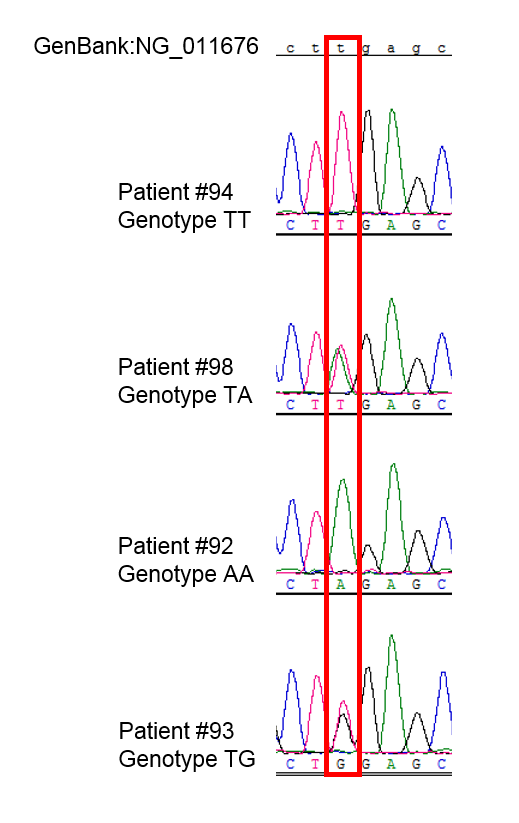


**Figure S1** The multiallelic polymorphism – rs965 (in red frame) found in the 5’UTR (untranslated region) of the *GH1* gene


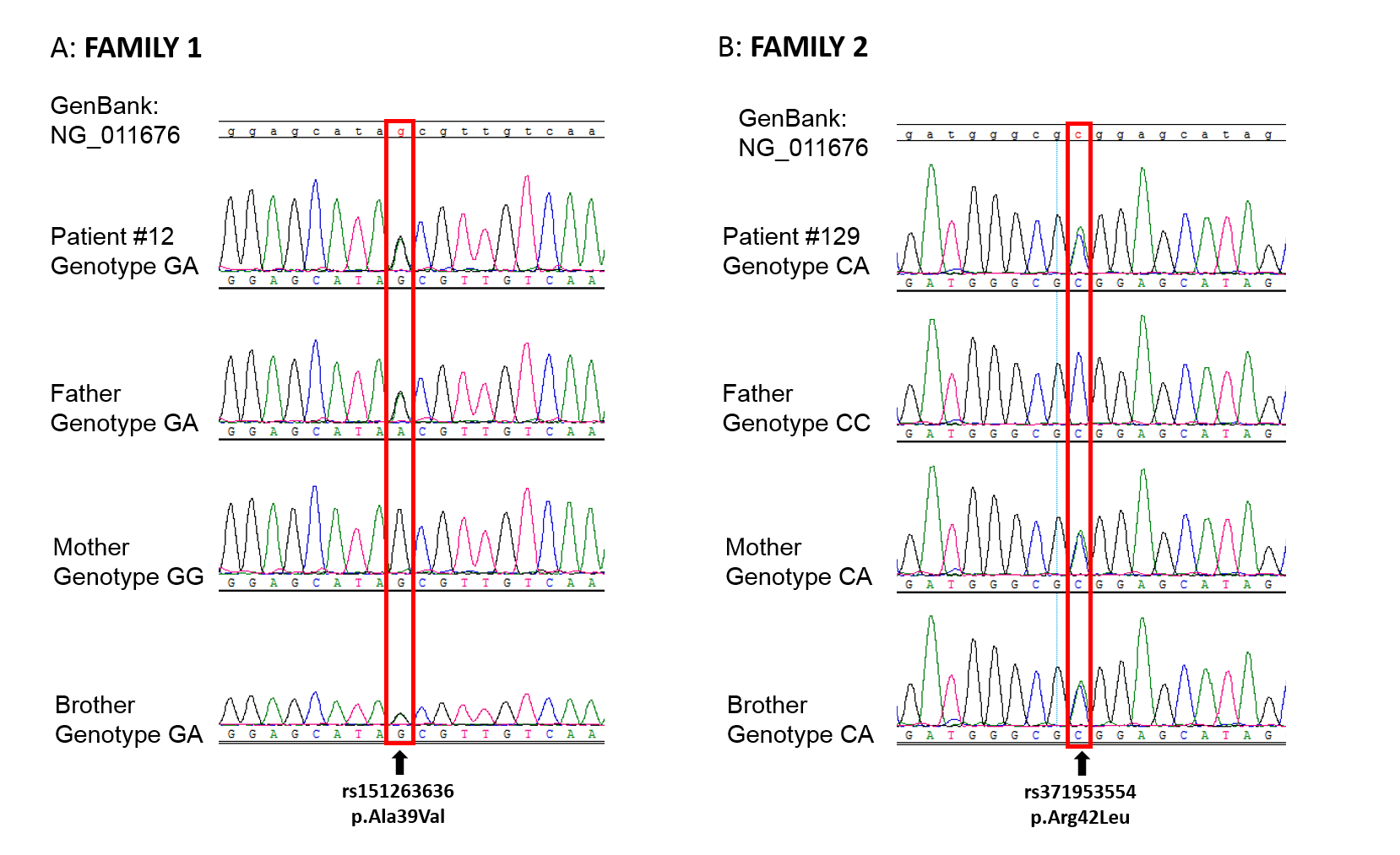


**Figure S2** The Sanger sequencing of the missense variants (rs151263636 and rs371953554) in exon 2 of *GH1* in the studied families
